# An Account of the Practice of One of the Physicians of the Westminster General Dispensary, and of the Western Dispensary, from the 20th of October, to the 20th of November

**Published:** 1806-12

**Authors:** S. Fothergill

**Affiliations:** Southampton Street, Strand


*An Account of the Practice of one of the Physicians of the Westminster General Dispensary, and of the Western Dispensary, from the 20th of October, to the 20th of November.*

| | |
|---|---|
| **ACUTE DISEASES.** | |
| Synochus - - - | 5 |
| Typhus - - - | 4 |
| Pleurisy - - - | 2 |
| Acute Rheumatism - | 7 |
| Cynanche Tonsillaris | 3 |
| Scarlatina - - | 5 |
| Parotitis - - - | 2 |
| Measles - - - | 4 |
| Catarrh - - - | 3 |
| Hooping-Cough - | 4 |
| Small-pox - - | 2 |
| Urticaria - - | 1 |
| Enteritis - - - | 1 |
| Cholera - - - | 1 |
| Acute Diseases of Infants | 15 |
| **CHRONIC DISEASES.** | |
| Rheumatism - - | 5 |
| Lumbago & Sciatica | 4 |
| Cephalalgia and Vertigo | 6 |
| Pulmonary Consumption | 5 |
| Asthma - - - | 2 |
| Cough and Dyspnœa | 20 |
| Pleurodyne - - | 3 |
| Hæmoptoe - - | 2 |
| Gastrodynia & Enterodynia - - - | 11 |
| Marasmus - - | 2 |
| Tabes Mesenterica - | 2 |
| Asthenia - - | 6 |
| Pyrosis - - - | 2 |
| Jaundice - - - | 2 |
| Diarrhœa - - - | 9 |
| Constipation & Vomiting | 1 |
| Dysentery - - | 1 |
| Dyspepsia - - | 8 |
| Dropsy - - - | 2 |
| Gravel and Dysuria | 3 |
| Paralysis - - - | 2 |
| Dysphagia - - | 1 |
| Epilepsy - - - | 1 |
| St. Vitus's Dance - | 1 |
| Locked Jaw - - | 1 |
| Angina Pectoris - | 1 |
| Diseased Prostate - | 1 |
| Worms - - - | 3 |
| Syphilis - - - | 3 |
| Gonorrhœa - - | 2 |
| Psora - - - | 3 |
| Tinea Capitis - - | 2 |
| Prurigo Senilis - | 1 |
| Scabies - - - | 2 |
| Chlorosis - - | [illegible] |
| Amenorrhœa - - | 3 |
| Menorrhagia - - | 2 |
| Leucorrhœa - - | 4 |
| Cancer of the Uterus - | 1 |

During the last month, and for some time previous, typhus fever has been very prevalent in many parts of London and Westminster and its fatal influence seems to be yet yet extending. When we see the affluent and those possessed of every comfort life can afford, surrounded by friends, and enjoying the best medical aid, fall victims to this dreadful malady, we are not to wonder that its ravages amongst the poor are widely extensive; indeed, with so much to favour the propagation and continuance of typhus fever amongst the immense population of London, it is rather matter of surprise that so few people are affected with a complaint, which in a city less cleanly and equally close and populous, would prove destructive beyond the power of calculation. Cleanliness is a virtue so eminently useful, that it cannot be too much encouraged amongst the lowest classes of the people; and if the Society for the Suppression of Vice, would devote part of their funds to cause the apartments of the poor to be annually white-washed, scoured, &c. and use some exertion in enforcing the necessity of cleanliness whilst they introduced a Bible into every house, I must think their labours would be not less beneficial to the community, than their efforts to destroy improper prints, punish obscenity, chastise iniquity, and chasten the writings of authors by incarcerating their persons.

The cases of typhus, noticed in a former report, have terminated favourably. In two of them, young men from the country, the subsequent debility continued so long and to such a degree, as to occasion considerable alarm for their ultimate recovery; the delirium was neither violent nor long continued; they were both extremely deaf, and one of them was in a state of fatuity for a considerable length of time after every symptom of fever had disappeared; he complained much of his head whilst sensible; and was on that account, besides my usual practice of early giving purgatives in this fever, repeatedly blistered, and I thought with good effect. Blisters are now much less used than formerly in the cure of typhus; and, I believe, they are of little use in mitigating the violence, or lessening the duration of the fever; where, however, there are local pains, particularly in the head, they have proved beneficial; Dr. Home, in his Clinical Experiments, observes "they are of the greatest utility in relieving the severe head-ach. Blisters applied to the temples, remove this symptom most successfully, without directly producing any good effect on the fever; though they may indirectly, by removing one cause of watchfulness and weakness."

Besides the measles, small-pox, hooping-cough, and scarlet-fever, with which so many young children have lately lately been attacked, several infants at the breast have suffered from cough and difficult breathing; in some, accompanied with pyrexia; in others with frequent green stools. In one family, four children became the subjects of measles before they had recovered from the effects of hooping-cough; two of them died, and the others were placed under my care in a state of great debility, affected with a hard troublesome cough, œdematous legs, and a disposition to general dropsy; small doses of steel, and calomel purges soon effected a beneficial change in their appearance, and they are now rapidly recovering. Many people are so alarmed at the name of an aperient, that some persuasion is requisite before they will submit to a practice, against which they are prejudiced, from the notion that it necessarily must reduce the strength and render debility weaker. Where strong doses and rough cathartics are avoided, and attention is paid to previous habits, I have not found this to be the case, and the amplest experience confirms the utility of aperient medicines, whilst modern practice seems favourable to their being more generally adopted, than they have been at any former period.

S. FOTHERGILL.

*Southampton Street, Strand, Nov.* 22, 1806.

